# Magnitude of Poor Sleep Hygiene Practice and Associated Factors among Medical Students in Ethiopia: A Cross-Sectional Study

**DOI:** 10.1155/2021/6611338

**Published:** 2021-02-15

**Authors:** Alemayehu Molla, Tirusew Wondie

**Affiliations:** ^1^Department of Psychiatry, College of Medicine and Health Sciences, Dilla University, Dilla, Ethiopia; ^2^Department of Psychiatry College of Medicine and Health Science, Debre Markos University, Debre Markos, Ethiopia

## Abstract

**Background:**

Good sleep hygiene plays an important role in human health. Medical students are notorious for insufficient and irregular sleep habits which are linked with students' learning abilities, poor academic performance, and poor interpersonal relationship which predispose them to mental illnesses. However, it has not been studied among medical students in Ethiopia.

**Method:**

This institution-based cross-sectional study was conducted among 576 undergraduate medical students selected by using a stratified sampling technique. Sleep hygiene (SHI) was assessed by a 13-item sleep hygiene questionnaire. Binary logistic regression was used to identify the potential determinants of poor sleep hygiene among undergraduate medical students. Variables with *p* values less than 0.05 were considered statistically significant, and the strength of the association was presented by adjusted odds ratio with 95% C.I.

**Result:**

The prevalence of poor sleep hygiene practice among undergraduate medical students was 48.1% (95% 43.7, 52.1). After adjusting for the possible confounders, being female (AOR = 1.53, 95% CI 1.03, 2.26), having depressive symptoms (AOR = 3.55, 95% CI 2.26, 5.59), with stress symptoms (AOR = 2.41, 95% CI 1.61, 3.60), and having anxiety symptoms (AOR = 2.2, 95% CI 1.42, 3.31) were associated with poor sleep hygiene practice at *p* value < 0.05.

**Conclusion:**

Almost half of the medical students had poor sleep hygiene practice. Routine screening of depressive and stress symptoms and education about sleep hygiene are warranted among medical students.

## 1. Introduction

Sleep hygiene depends on behaviors and environmental conditions [1] that are aimed at ensuring good quality sleep and is important to avoid sleep disorders [2]. It is a one component of Cognitive-Behavioral Therapy for Insomnia (CBT-I) to treat chronic insomnia [2–5]. People who get enough sleep have more energy and better cognitive function and performance throughout the day [6]. Poor sleep quality among medical students has significant impact on mental and physical health, leading to problem of drinking and suicidal thought which adversely affects academic performance and influences the community [7–9]. And also, it affects their future work performance as practitioners and the health care system [10].

WHO report showed that about 27% of people suffer from sleep problems worldwide [11]. The rate of sleep difficulties reported in developed nations like USA also showed that 50 to 70 million people are chronically suffering from sleep disorders [12, 13]. Students are notorious for insufficient and poor quality sleep and for irregular sleep habits, such as sleeping less during the week [14–19]. Prevalence of poor sleep is twofold high among university than general population as a result of shift sleep–wake cycle due to their study and work schedules [10, 20]. Poor sleep hygiene practices are linked with students' abilities of learning and academic performance [21, 22].

Medical study is one of the most stressful fields, and poor sleep is more common among medical students than nonmedical students [23]. As studies reported, medical students have a more stressful academic program related to long duration of study year and overnight clinical duties which affect their habits of sleep and result in sleep difficulties [24–26]. A study conducted in developing countries showed that 32.5%-76% of medical students suffer from poor sleep quality [27, 28].

Both the quality and quantity of sleep affect student's ability to cope with emotional challenges [29]. Conversely, sleep deprivation and sleep disturbances trigger negative emotional reactivity and diminish the effect of positive emotions [29]. For these reasons, practices and behaviors that promote consistent and uninterrupted sleep are important to improve sleep quality and increase daytime alertness [30].

Sleep hygiene includes different practices and behaviors like limiting daytime naps to less than 30 minutes, regular exercise, limited exposure to phones, consistency on sleep schedule, avoidance of stimulants close to bedtime, and avoiding eating large meals late in the evening and similar [30].

Factors like coffee or tea; excessive use of social media; use of drugs; medical problems; having depressive, anxiety, and stress symptoms; academic performance; and gender were associated with poor sleep hygiene practice in different studies [10, 19, 31].

Despite the high prevalence of poor sleep hygiene practice among medical students, as per the investigators' knowledge, there is no specific study among medical students in Ethiopia. Therefore, this study was intended to assess the magnitude of poor sleep hygiene practice and associated factors among medical students in Ethiopia.

## 2. Methods and Materials

### 2.1. Study Design and Setting

An institutional-based cross-sectional study was conducted from May 1-June 8, 2019. The study was conducted at Tikur Anbessa Specialized Hospital which is found in Addis Ababa, and it is the largest referral hospital in the country with more than 700 beds. The hospital addresses students coming from all parts of Ethiopia for medical study. A total of 20100 students are available and 1647 undergraduate medical student's studies in the hospital.

### 2.2. Study Population

The study participants were all undergraduate medical students at Tikur Anbessa Specialized Hospital. Students who were severely ill with difficulty of communication during the study period were excluded.

### 2.3. Sample Size Determination and Sampling Procedure

The sample size was determined with the following assumptions: margin of error 4, at 95% CI, proportion 61.4%, which was taken from Sudan and nonresponse rate of 10%. The final calculated sample size was 626. Stratified sampling technique was used to select samples. To assure representativeness of the sample, proportional allocation was done to the respective class year or strata. The list of students was obtained from college registrar, and computer-generated simple random sampling method was used to select each study participants from their respective group.

### 2.4. Data Collection and Tools

Data was collected by using interviewer-administered structured and pretested questionnaires. It was collected by three BSc psychiatry nurses and supervised by one mental health professional. Sociodemographic characteristics were collected by semistructured questionnaires; also, current uses of substance (alcohol, cigarette, and khat) were assessed by using of a specific substance for nonmedical purpose in the last three months.

The outcome variable Sleep Hygiene Index (SHI) was assessed by a 13-item sleep hygiene questionnaire. Each item is rated on a five-point scale ranging from 0 (never) to 4 (always). Total scores range from 0 to 52, with a higher score representing poor sleep hygiene. SHI has shown adequate reliability and validity [32, 33]. Social support was measured using Oslo 3-item social support scale (OSS-3) having poor social support, a score of “3-8”; intermediate social support, a score of “9-11”; and strong social support, a score of “12-14” [34]. Depression, anxiety, and stress symptoms were measured using Lovibond and Lovibond's short version of the depression anxiety stress scale (DASS-21). The questionnaires explain the experience of the items in the past week, and each item is scored from 0 (did not apply to me at all) to 3 (applied to me very much). Finally, the values obtained were multiplied by 2 [35]. The reliability of these instruments in the current study is checked using the Cronbach *α*, and it is 91% for depression, 72.2%for anxiety, and 88.2% for stress components.

### 2.5. Statistical Analysis

The data were entered into the computer using EPI Data version 3.1 and exported to statistical package for social science (SPSS) version 20. Associations of sleep hygiene practice and associated factors were identified using logistic regression. Following each bivariate regression, a multivariable logistic regression model was constructed. Variables with *p* value below 0.05 were considered statistically significant, and the strength of associations was determined using adjusted odds ratio (AOR) at 95% CI.

## 3. Results

### 3.1. Sociodemographic Characteristics of Respondents

A total of 576 participants were included in the study, and the response rate was 92%. The mean age (±SD) of the respondents was 21.5 (±2.4), with age ranging from 18 to 28 years and about 310 (53.8%) of participants were males. Educational status of the students showed that about 128 (22%) of respondents were fourth year. Regarding marital status, majorities of the respondents were single (94.1%) and the mean of cumulative grade was 2.81 ± 0.59 (Table 1).

### 3.2. Clinical, Behavioral, and Social Factors of the Respondents

Among participants, 369 (64.1%) and 322 (55.9%) had depressive and stress symptoms, respectively. Regarding substance-related factors, about 236 (41%) of the respondents use alcohol currently within three months and 280 (48.6%) of the participants reported that they had poor social support (Table 2).

### 3.3. Magnitude of Poor Sleep Hygiene among Medical Students

Overall, 277 [48.1% (95% CI, 43.7, 52.1)] of students have poor sleep hygiene practice. Regarding component results of sleep hygiene practice, a relatively large numbers (18.1%) of participants always use their bed other than sleeping for watching television, reading, and eating food followed by 15.5% of respondents think, plan and, worry in bed. Among participants, 8.5% of them always use coffee before their bed time and about 15.3% of respondents always go to bed at different time. Poor sleep hygiene is also high among participants with age category of ≤21 years (55%).

### 3.4. Factors Associated with Sleep Hygiene Practice

In bivariate binary logistic analysis variables, being female, age range ≤21 years, current use of alcohol, having depressive anxiety, and stress symptoms were associated with poor sleep hygiene practice at *p* value less than 0.25.

But, in multivariable logistic regression, variables, being female, having depressive, stress, and anxiety symptoms were statistically significant with poor sleep hygiene practice at *p* value less than 0.05.

The odds of having poor sleep hygiene practices among female students was 1.53 times higher as compared to their counter parts (AOR = 1.53, 95% CI, (1,03, 2.26). The odds of having poor sleep hygiene practice among respondents with stress symptoms was 2.41 times higher as compared to those without stress (AOR = 2.41, 95% CI, 1.61, 3.60).

Regarding depression, participants with depressive symptoms were 3.55 times more likely to have poor sleep hygiene practice as compared with respondents who had no depression (AOR = 3.55, 95% CI, 2.26, 5.59). The presence of anxiety symptom was also another factor which associated with poor sleep hygiene practice. Participants with anxiety symptoms were 2.2 times more likely to develop poor sleep hygiene practice as compared with their counter parts (AOR = 2.2, 95% CI (1.42, 3.31)) (Table 3).

## 4. Discussion

In this study, the prevalence of poor sleep hygiene practice was 48.1% (95% 43.7, 52.1), which is in line with the study conducted in Southern Universities 40-50% [36], Brazil 51.5% [37], US 50.9% [38], and Hong Kong [14], showing that large numbers of participants did not practice sleep hygiene components like using of bed for night sleep purpose and avoidance of coffee and other stimulants during bed time. However, the result of the present study is higher than that of the study reported in Nigeria 32% [27] and Pakistan Karachi 39.5%. On the other hand, this study is lower than the previous studies done in Iran 57.5% [39], Saudi Arabia 70.4%-76% [28, 40, 41], India 72.9% [42], and Pakistani 77% [23]. The possible reasons for the variability could be due to differences in sampling technique, which was consecutive sampling technique used in Pakistan Karachi. Study population difference also might have its contribution for variation since a study conducted in Nigeria used only 5th and 6th year students and in Central India, first and second year students were the study participants. Our study included all medical students which might be attributed to the high number of lectures and study load during the early preclinical years, and those students may not adequately adapt to such high-load classes after studying at secondary schools. In our country, medical students have wider academic program, long duration of study year, and clinical duties including overnight duties which might affect their habits of maintaining good sleep hygiene practice. Another possible reason might be related to differences in cultural habits, socioeconomic status, and nature of universities from different countries.

Regarding factors associated with poor sleep hygiene practice, being female was strongly associated with poor sleep hygiene practice and the finding is supported with previous studies conducted by Cheng et al. [43] and this is also consistent with other studies conducted in different countries [44–47]. But it is contrary to a study conducted in Iraq among medical students of Qazvin University. The possible reason might be due to the fact that females physiologically need more time for sleep [44, 48].

There is also significant association between having stress symptoms and poor sleep hygiene practice among medical students. This is similar to the finding of study conducted in Pakistan [23] and Saudi Arabia [28]. Physiologically, studies have found that sleep and stress are closely link to the hypothalamus-pituitary-adrenal axis [49, 50]. Acute stress decreases slow wave and rapid eye movement and increases sleep deprivation [50].

In this study, the odds of having poor sleep hygiene practice among respondents with depressive symptoms was 3.55 times higher as compared to their counterparts. This finding coincides with results from Hong Kong [14], Virginia [19], and Saudi Arabia [41]. The possible reason might be due to decreased amount of neurotransmitter serotonin and results in diminished cognitive performance that affects normal sleep pattern. Finally, having anxiety symptom was another factor associated with poor sleep hygiene practice among medical students. This finding was supported by a study conducted in southern universities [36] and Lithuanian medical students [51]. The possible explanation for this might be due to fear which makes it harder to fall asleep. In addition, sleep deprivation can worsen anxiety, spurring a negative cycle involving insomnia and anxiety disorders [52].

Evidences showed that a cognitive behavioral therapy for insomnia (CBT-I) is important in improving sleep problem among college students [53–55]. The cognitive behavioral therapy for insomnia (CBT-I) is a structured and evidence-based technique to reduce the symptoms of insomnia by focusing on the connections between the way we think, the things we do, and how we sleep. The trained CBT-I providers can identify and challenge thoughts, feelings, and behaviors of individuals with sleep problem [56, 57]. Studies also showed that sleep education intervention is important in improving and maintaining sleep hygiene practice and sleep quality. Improvements were shown in self-reported sleep behaviors including stopping electronics earlier, waking earlier during the week, keeping a more regular sleep schedule, and napping less [38, 58, 59].

Different studies showed bidirectional relationship between insomnia, anxiety, and depression. This suggests that insomnia, anxiety, and depression are intertwined over time [60–62]. As a result, the potential interactive mechanisms and therapeutic strategy for depression and anxiety in sleep disturbance need bidirectional consideration.

## 5. Conclusion

Approximately half of the participants had poor sleep hygiene practice. Being female and having stress, depressive, and anxiety symptoms were found to be significant predictors. Institution-based academic counseling center focusing on student's study skill and coping with their stressful environment is crucial. Moreover, it is better to educate medical students about proper sleep hygiene and the consequences of poor sleep hygiene practices. It is important to give cognitive behavioral therapy for insomnia (CBT-I) for students who are female, with age range ≤ 21 years and having depressive, anxiety, and stress symptoms. It would have been better to conduct prospective studies to investigate the cause and effect relationship of risk factors of poor sleep hygiene practice.

### 5.1. Limitation of the Study

The nature of the study design might not establish temporal relationship between outcome and independent variables in this study.

## Figures and Tables

**Table 1:** Sociodemographic characteristics among undergraduate medical students in Ethiopia, 2019 (*N* = 576).

Variables		Frequency	Percentage (%)
Sex	Female	266	45.2
	Male	310	53.8
Age	≤21	294	51
	>21	282	49
Marital status	Single	542	94.1
	Married	34	5.9
Year of study	First year	112	19.4
	Second year	85	14.8
	Third year	108	18.8
	Fourth year	128	22.2
	Fifth year	69	12.0
	Sixth year	74	12.8
Cumulative grade point	Mean + standard deviation		2.81 ± 0.59

**Table 2:** Clinical, behavioral, and social factors among undergraduate medical students in Ethiopia, 2019 (*N* = 576).

Variables	Frequency	Percentage (%)
Anxiety symptoms		
Yes	315	54.7
No	261	45.3
Stress symptoms		
Yes	322	55.9
No	254	44.1
Depression symptoms		
Yes	369	64.1
No	207	35.9
Current alcohol use		
Yes	236	41
No	340	59
Current cigarette use		
No	544	94.4
Yes	32	5.6
Current khat use		
No	518	89.9
Yes	58	10.1
Social support		
Poor	280	48.6
Moderate	174	30.2
Strong	122	21.2

**Table 3:** Logistic regression showing association between factors and sleep hygiene practice among undergraduate medical students in Ethiopia, 2019 (*N* = 576).

Explanatory variables	Sleep hygiene practice		COR 95% CI)	AOR (95% CI)
	Poor	Good		
	(*n* = 357)	(*n* = 219)		
	*n*%	*n*%		
Sex				
Female	107	123	1.11 (0.79, 1.55)	1.53 (1.03, 2.26)^∗^
Male	170	176	1.00	1.00
Age				
≤21	133	166	1.73 (1.25, 2.41)	1.17 (0.78, 1.76)
>21	116	161	1.00	1.00
Current alcohol use				
Yes	124	112	1.35 (0.97, 1.89)	1.38 (0.92, 1.2.08)
No	153	187	1.00	1.00
Current khat use				
Yes	26	32	0.89 (0.50, 1.49)	0.90 (0.47, 1.71)
No	251	267	1.00	1.00
Depressive symptoms				
Yes	223	136	6.35 (4.28, 9.42)	3.55 (2.26, 5.59)^∗^
No	44	163	1.00	1.00
Stress symptoms				
Yes	204	118	4.29 (3.01, 6.11)	2.41 (1.61, 3.60)^∗^
No	73	181	1.00	1.00
Anxiety symptoms				
Yes	199	116	4.03 (2.28, 5.71)	2.2 (1.42, 3.31)^∗^
No	78	183	1.00	1.00

N.B. 1.00 reference; ^∗^p value less than 0.05.

## Data Availability

The raw data included in the manuscript is available and can be accessed from the corresponding author.

## References

[1] Berry R. B., Budhiraja R., Gottlieb D. J. (2012). Rules for scoring respiratory events in sleep: update of the 2007 AASM manual for the scoring of sleep and associated events: deliberations of the sleep apnea definitions task force of the American Academy of Sleep Medicine. *Journal of Clinical Sleep Medicine*.

[2] Perlis M. L., Aloia M., Kuhn B. (2010). *Behavioral Treatments for Sleep Disorders: A Comprehensive Primer of Behavioral Sleep Medicine Interventions*.

[3] Voinescu B. I., Szentagotai-Tatar A. (2015). Sleep hygiene awareness: its relation to sleep quality and diurnal preference. *Journal of molecular psychiatry*.

[4] Perlis M. L., Jungquist C., Smith M. T., Posner D. (2006). *Cognitive behavioral treatment of insomnia: a session-by-session guide, vol. 1*.

[5] Morin C. M., Bootzin R. R., Buysse D. J., Edinger J. D., Espie C. A., Lichstein K. L. (2006). Psychological and behavioral treatment of insomnia: update of the recent evidence (1998–2004). *Sleep*.

[6] Åkerstedt T., Hume K., Minors D., Waterhouse J. (1997). Good sleep—its timing and physiological sleep characteristics. *Journal of Sleep Research*.

[7] Mume C. O., Olawale K. O., Osundina A. F. (2011). Excessive daytime sleepiness, nocturnal sleep duration and psychopathology among Nigerian university students. *South African Journal of Psychiatry*.

[8] Kenney S. R., LaBrie J. W., Hummer J. F., Pham A. T. (2012). Global sleep quality as a moderator of alcohol consumption and consequences in college students. *Addictive Behaviors*.

[9] Nadorff M. R., Nazem S., Fiske A. (2011). Insomnia symptoms, nightmares, and suicidal ideation in a college student sample. *Sleep*.

[10] Azad M. C., Fraser K., Rumana N. (2015). Sleep disturbances among medical students: a global perspective. *Journal of Clinical Sleep Medicine*.

[11] Yang C.-M., Wu C.-H., Hsieh M.-H., Liu M.-H., Lu F.-H. (2003). Coping with sleep disturbances among young adults: a survey of first-year college students in Taiwan. *Behavioral Medicine*.

[12] Stranges S., Tigbe W., Gómez-Olivé F. X., Thorogood M., Kandala N.-B. (2012). Sleep problems: an emerging global epidemic? Findings from the INDEPTH WHO-SAGE study among more than 40,000 older adults from 8 countries across Africa and Asia. *Sleep*.

[13] Altevogt B. M., Colten H. R. (2006). *Sleep Disorders and Sleep Deprivation: An Unmet Public Health Problem*.

[14] Suen L., Tam W., Hon K. (2010). Association of sleep hygiene-related factors and sleep quality among university students in Hong Kong. *Hong Kong Medical Journal*.

[15] Hicks R. A., Fernandez C., Pellegrini R. J. (2016). The changing sleep habits of university students: an update. *Perceptual and Motor Skills*.

[16] Pallos H., Gergely V., Yamada N., Miyazaki S., Okawa M. (2007). The quality of sleep and factors associated with poor sleep in Japanese graduate students. *Sleep and Biological Rhythms*.

[17] Taylor D. J., Gardner C. E., Bramoweth A. D. (2011). Insomnia and mental health in college students. *Behavioral Sleep Medicine*.

[18] Voinescu B. I., Szentagotai A., David D. (2012). Sleep disturbance, circadian preference and symptoms of adult attention deficit hyperactivity disorder (ADHD). *Journal of Neural Transmission*.

[19] Lund H. G., Reider B. D., Whiting A. B., Prichard J. R. (2010). Sleep patterns and predictors of disturbed sleep in a large population of college students. *Journal of Adolescent Health*.

[20] Brown F. C., Soper B., Buboltz W. C. (2001). Prevalence of delayed sleep phase syndrome in university students. *College Student Journal*.

[21] BaHammam A. S., Alaseem A. M., Alzakri A. A., Almeneessier A. S., Sharif M. M. (2012). The relationship between sleep and wake habits and academic performance in medical students: a cross-sectional study. *BMC Medical Education*.

[22] Curcio G., Ferrara M., De Gennaro L. (2006). Sleep loss, learning capacity and academic performance. *Sleep Medicine Reviews*.

[23] Waqas A., Khan S., Sharif W., Khalid U., Ali A. (2015). Association of academic stress with sleeping difficulties in medical students of a Pakistani medical school: a cross sectional survey. *PeerJ*.

[24] Abdulghani H. M., Alrowais N. A., Bin-Saad N. S., Al-Subaie N. M., Haji A. M., Alhaqwi A. I. (2012). Sleep disorder among medical students: relationship to their academic performance. *Medical teacher*.

[25] Sahraian A., Javadpour A. (2010). Sleep disruption and its correlation to psychological distress among medical students. *Shiraz E Medical Journal*.

[26] Wong J., Patil N., Beh S. (2009). Cultivating psychological well-being in Hong Kong's future doctors. *Medical Teacher*.

[27] James B. O., Omoaregba J. O., Igberase O. O. (2011). Prevalence and correlates of poor sleep quality among medical students at a Nigerian university. *Annals of Nigerian Medicine*.

[28] Almojali A. I., Almalki S. A., Alothman A. S., Masuadi E. M., Alaqeel M. K. (2017). The prevalence and association of stress with sleep quality among medical students. *Journal of epidemiology and global health*.

[29] Vandekerckhove M., Cluydts R. (2010). The emotional brain and sleep: an intimate relationship. *Sleep Medicine Reviews*.

[30] Shriane A. E., Ferguson S. A., Jay S. M., Vincent G. E. (2020). Sleep hygiene in shift workers: a systematic literature review. *Sleep Medicine Reviews*.

[31] Haseli-Mashhadi N., Dadd T., Pan A., Yu Z., Lin X., Franco O. H. (2009). Sleep quality in middle-aged and elderly Chinese: distribution, associated factors and associations with cardio-metabolic risk factors. *BMC Public Health*.

[32] Mastin D. F., Bryson J., Corwyn R. (2006). Assessment of sleep hygiene using the Sleep Hygiene Index. *Journal of Behavioral Medicine*.

[33] Ozdemir P. G., Boysan M., Selvi Y., Yildirim A., Yilmaz E. (2015). Psychometric properties of the Turkish version of the Sleep Hygiene Index in clinical and non-clinical samples. *Comprehensive Psychiatry*.

[34] Dalgard O. (2009). Social support-consequences for individual and society. *Journal of EUPHIX, EUphact Bilthoven: RIVM, EUphact\Determinants of health\Environment\Social support*.

[35] Szabó M. (2010). The short version of the Depression Anxiety Stress Scales (DASS-21): factor structure in a young adolescent sample. *Journal of Adolescence*.

[36] Felix V. A., Campsen N. A., White A., Buboltz W. C. (2017). College students’ prevalence of sleep hygiene awareness and practices. *Advances in Social Sciences Research Journal*.

[37] Pacheco J. P., Giacomin H. T., Tam W. W. (2017). Mental health problems among medical students in Brazil: a systematic review and meta-analysis. *Brazilian Journal of Psychiatry*.

[38] Brick C. A., Seely D. L., Palermo T. M. (2010). Association between sleep hygiene and sleep quality in medical students. *Behavioral Sleep Medicine*.

[39] Yazdi Z., Loukzadeh Z., Moghaddam P., Jalilolghadr S. (2016). Sleep hygiene practices and their relation to sleep quality in medical students of Qazvin University of Medical Sciences. *Journal of Caring Sciences*.

[40] Siddiqui A. F., Al-Musa H., Al-Amri H., Al-Qahtani A., Al-Shahrani M., Al-Qahtani M. (2016). Sleep patterns and predictors of poor sleep quality among medical students in King Khalid University, Saudi Arabia. *The Malaysian journal of medical sciences: MJMS*.

[41] Ibrahim N., Badawi F., Mansouri Y., Ainousa A., Jambi S. (2017). Sleep quality among medical students at King Abdulaziz University: a cross-sectional study. *Journal of Community Medicine and Health Education*.

[42] Shad R., Thawani R., Goel A. (2015). Burnout and sleep quality: a cross-sectional questionnaire-based study of medical and non-medical students in India. *Cureus*.

[43] Cheng S. H., Shih C.-C., Lee I. H. (2012). A study on the sleep quality of incoming university students. *Psychiatry Research*.

[44] Medeiros A. L. D., Mendes D. B., Lima P. F., Araujo J. F. (2010). The relationships between sleep-wake cycle and academic performance in medical students. *Biological Rhythm Research*.

[45] Sweileh W. M., Ali I. A., Sawalha A. F., Abu-Taha A. S., Sa'ed H. Z., Al-Jabi S. W. (2011). Sleep habits and sleep problems among Palestinian students. *Child and Adolescent Psychiatry and Mental Health*.

[46] Aghajanloo A., Haririan H., Ghafourifard M., Bagheri H., Ebrahimi S. M. (2011). Sleep quality of students during final exams in Zanjan University of Medical Sciences. *Modern Care Journal*.

[47] Horton L. E., Tarbox S. I., Olino T. M., Haas G. L. (2015). Trajectories of premorbid childhood and adolescent functioning in schizophrenia-spectrum psychoses: a first-episode study. *Psychiatry Research*.

[48] Abbasi Z. Y. Z. L. M., Mahmoodi M. Z. M. (2013). *Prevalence of insomnia and its relation with academic performance of medical students in Qazvin University of Medical Sciences*.

[49] Kashani M., Eliasson A., Vernalis M. (2011). Perceived stress correlates with disturbed sleep: a link connecting stress and cardiovascular disease. *Stress*.

[50] Van Reeth O., Weibel L., Spiegel K., Leproult R., Dugovic C., Maccari S. (2000). Interactions between stress and sleep: from basic research to clinical situations. *Sleep medicine reviews*.

[51] Preišegolavičiūtė E., Leskauskas D., Adomaitienė V. (2010). Associations of quality of sleep with lifestyle factors and profile of studies among Lithuanian students. *Medicina*.

[52] Deng J., Zhou F., Hou W. (2020). The prevalence of depression, anxiety, and sleep disturbances in COVID-19 patients: a meta-analysis. *Annals of the New York Academy of Sciences*.

[53] Trockel M., Manber R., Chang V., Thurston A., Tailor C. B. (2011). An e-mail delivered CBT for sleep-health program for college students: effects on sleep quality and depression symptoms. *Journal of Clinical Sleep Medicine*.

[54] Castronovo V., Galbiati A., Sforza M. (2018). Long-term clinical effect of group cognitive behavioral therapy for insomnia: a case series study. *Sleep Medicine*.

[55] Baglioni C., Altena E., Bjorvatn B. (2020). The European academy for cognitive behavioural therapy for insomnia: an initiative of the European insomnia network to promote implementation and dissemination of treatment. *Journal of Sleep Research*.

[56] Koffel E., Bramoweth A. D., Ulmer C. S. (2018). Increasing access to and utilization of cognitive behavioral therapy for insomnia (CBT-I): a narrative review. *Journal of General Internal Medicine*.

[57] Dewald-Kaufmann J., de Bruin E., Michael G. (2019). Cognitive behavioral therapy for insomnia (CBT-i) in school-aged children and adolescents. *Sleep Medicine Clinics*.

[58] Hershner S., O'brien L. M. (2018). The impact of a randomized sleep education intervention for college students. *Journal of Clinical Sleep Medicine*.

[59] Perlis M. L., Lichstein K. L. (2003). *Treating Sleep Disorders: Principles and Practice of Behavioral Sleep Medicine*.

[60] Sivertsen B., Salo P., Mykletun A. (2012). The bidirectional association between depression and insomnia: the HUNT study. *Psychosomatic Medicine*.

[61] Jansson-Fröjmark M., Lindblom K. (2008). A bidirectional relationship between anxiety and depression, and insomnia? A prospective study in the general population. *Journal of Psychosomatic Research*.

[62] Alvaro P. K., Roberts R. M., Harris J. K. (2013). A systematic review assessing bidirectionality between sleep disturbances, anxiety, and depression. *Sleep*.

